# Efficacy and Neurophysiological Mechanisms of 10 Hz Repetitive Transcranial Magnetic Stimulation for Post-Stroke Dysphagia: A Randomized Controlled Trial

**DOI:** 10.31083/RN49912

**Published:** 2026-04-22

**Authors:** Bangqiang Hou, Ke Pan, Rong Zhang, Xiaojuan Chen, Yizheng Li, Yinxu Wang, Yulei Xie

**Affiliations:** ^1^Department of Rehabilitation Medicine, Affiliated Hospital of North Sichuan Medical College, 637000 Nanchong, Sichuan, China; ^2^Department of Radiology, Affiliated Hospital of North Sichuan Medical College, 637000 Nanchong, Sichuan, China; ^3^Department of Rehabilitation Medicine, Qinghai Provincial People's Hospital, 810000 Xining, Qinghai, China

**Keywords:** transcranial magnetic stimulation, stroke, post-stroke dysphagia, randomized controlled trial

## Abstract

**Background:**

Post-stroke dysphagia (PSD) is a common and serious complication, yet conventional rehabilitation therapies have limited efficacy. Repetitive transcranial magnetic stimulation (rTMS) is a promising treatment, but its optimal intervention strategy remains undetermined.

**Methods:**

Seventy-five PSD patients were randomly assigned in a 1:1:1 ratio to the sham rTMS group, affected rTMS group and bilateral rTMS group. All groups received the corresponding rTMS intervention and conventional rehabilitation therapy. Swallowing function was assessed at baseline (T0) and after treatment (T1) using the standardized swallowing assessment (SSA), penetration-aspiration scale (PAS), yale pharyngeal residue severity rating scale (YPR-SRS), and suprahyoid motor evoked potentials (MEP). Adverse reactions and dropouts were recorded.

**Results:**

After treatment, the SSA scores of all three groups were significantly improved. Bilateral rTMS showed significantly greater improvement in SSA and a higher treatment response rate (77.27%) compared to both the sham group and the affected rTMS group (*p* < 0.001). Mixed-effects model and intention-to-treat analyses both supported the optimal efficacy of bilateral rTMS (interaction effect *p* < 0.01). Regarding swallowing safety (PAS), the bilateral rTMS group's score was significantly lower than that of the sham group (*p* = 0.017). In terms of pharyngeal residue clearance (YPR-SRS), the bilateral rTMS group showed significantly greater improvement in the piriform sinuses compared to the other two groups, and superior improvement in the vallecula compared to the sham group (*p* < 0.05). After treatment, MEP amplitudes increased in all groups. Notably, only the bilateral rTMS group not only significantly increased MEP amplitudes on both sides (*p* < 0.01) but also significantly shortened the latency on the contralesional side (*p* = 0.046). The bilateral rTMS group achieved a “large effect size” in improving SSA scores, increasing MEP amplitudes, and shortening latency on the contralesional side, with the SSA effect size (D = 2.339) far exceeding that of the other groups. All treatment regimens were well-tolerated, with only 5 cases of transient scalp discomfort reported and no serious adverse events.

**Conclusions:**

Conventional rehabilitation combined with 10 Hz rTMS targeting the swallowing cortex can effectively improve swallowing function in PSD patients. Bilateral rTMS is a superior strategy. Its therapeutic advantage may stem from the synergistic modulation of bilateral cortical excitability and neural conduction efficiency, providing a better multi-target neuromodulation option for clinical practice.

**Clinical Trial Registration:**

No: ChiCTR2300068730. https://www.chictr.org.cn/showproj.html?proj=182568.

## 1. Introduction

Dysphagia is one of the common complications following stroke, with an incidence rate ranging from 21% to 64% [1]. Post-stroke dysphagia (PSD) can lead to dehydration, malnutrition, aspiration and lung infections, in severe cases, can be life-threatening, imposing a heavy burden on both patients and their families [2]. Current conventional treatment options for PSD include swallowing exercises, physical factor therapy based on electrical swallowing stimulation, traditional Chinese medicine therapy, posture training and dietary modifications. Although these methods have some efficacy, they often encounter limitations. Finding ways to rapidly improve swallowing function, reduce various complications and enhance the quality of life for patients remains a key focus and challenge [3, 4].

Repetitive transcranial magnetic stimulation (rTMS) is one of the commonly used and well-supported neuromodulation techniques in stroke rehabilitation [5, 6]. In recent years, the use of rTMS in patients with PSD has increased significantly, demonstrating promising clinical potential [5, 7]. The therapeutic mechanisms of rTMS for PSD are currently understood through several theoretical models: The interhemispheric competition theory posits that post-stroke interhemispheric inhibition via the corpus callosum is disrupted, leading to reduced excitability in the affected hemisphere and increased inhibition from the unaffected hemisphere, which is considered a key contributor to swallowing dysfunction [8]. According to this model, applying high-frequency rTMS to the affected hemisphere can help restore excitability balance between the two hemispheres, thereby improving swallowing function [9, 10]. The compensation theory of the unaffected hemisphere suggests that when the affected hemisphere is severely damaged and corticobulbar pathways are compromised, activating compensatory pathways in the unaffected hemisphere becomes crucial for functional recovery [11]. Furthermore, the dual-balance model emphasizes that simultaneous modulation of excitability in both hemispheres may optimize neural network function more effectively than unilateral stimulation alone [12]. Beyond targeting the cerebral cortex, modulating the cerebellum and its functional connections with cortical regions involved in swallowing has emerged as a new research direction, aiming to improve swallowing function by influencing cortical–cerebellar circuits [13, 14].

Although the aforementioned theories provide a basis for different stimulation strategies, there is still no consensus on which strategy is optimal. While preliminary evidence from previous studies and the present study suggests that high-frequency rTMS targeting bilateral cerebral cortices may be a superior approach, this hypothesis still requires validation through rigorously designed, high-quality clinical trials [13, 15, 16, 17, 18].

Therefore, this study adopted a high-frequency (10 Hz) rTMS protocol with multi-target combined modulation. This protocol is based on prior evidence indicating that high-frequency rTMS (≥5 Hz) can induce long-lasting enhancement of cortical excitability, thereby more effectively promoting neuroplasticity in swallowing-related neural networks [9, 13, 18]. Although 5 Hz stimulation has also been applied in cortical modulation, the present study aimed to explore the potential synergistic and enhanced effects of a higher stimulation frequency under a bilateral synchronous stimulation paradigm, with the goal of surpassing the limitations of current treatment strategies.

## 2. Methods and Objectives

### 2.1 Study Subjects

Patients with PSD were recruited from the Affiliated Hospital of North Sichuan Medical College between March 2023 and October 2025. All participants met the predefined inclusion and exclusion criteria. Before the commencement of the study, written informed consent was obtained from each patient or their legal guardian/immediate family member, ensuring full comprehension of the study’s purpose, procedures, potential risks, and benefits. The trial protocol was approved by the Medical Ethics Committee of the Affiliated Hospital of North Sichuan Medical College (approval numbers: 2023ER031-1) and registered in the Chinese Clinical Trial Registry (registration number: ChiCTR2300068730).

### 2.2 Study Design

This study was designed as a single-center, single-blind, randomized controlled trial with an integrated multimodal evaluation system to comprehensively examine intervention effects and underlying mechanisms. Assessment modalities included the standardized swallowing assessment (SSA), fiberoptic endoscopic evaluation of swallowing (FEES), and suprahyoid muscle motor evoked potentials (MEP). The study aimed to systematically elucidate the intervention’s pathways and neuromodulatory mechanisms from both behavioral and neurophysiological perspectives, providing a more precise scientific basis for the rehabilitation of patients with PSD.

Block randomization was employed, with an independent statistician not involved in patient recruitment or assessment generating a random sequence using computer software. The sequence allocated patients in a 1:1:1 ratio to the sham group, affected rTMS group and bilateral rTMS group. Allocation concealment was ensured using sequentially numbered, opaque, sealed envelopes containing the group assignment. Eligible patients who provided informed consent were assigned to a group by a study coordinator who opened the corresponding envelope in sequential order, thereby determining the group allocation and treatment protocol. Blinding was applied to both patients and outcome assessors; only the treating operator was aware of the group assignment. All outcome measures were assessed at baseline (before treatment) and immediately post-intervention by the same therapist, who was blinded to group allocation and not involved in the intervention. This assessor received standardized training prior to the trial to ensure consistency in evaluation. Throughout the trial, adverse events (including epilepsy, headache, dizziness, syncope, dyspnea, scalp/neck skin redness, tinnitus, etc.) and participant dropout were recorded in detail to comprehensively evaluate treatment safety and tolerability.

This trial employed a random grouping design, consisting of a total of three groups. The significance level (α) was set at 0.05, and the statistical power (1–β) was set at 0.9. Based on effect sizes, standard deviations, and preliminary experimental data reported in previous literature, PASS 15 software (version 15.0, NCSS, LLC, Kaysville, UT, USA) was used to estimate the sample size, resulting in a requirement of at least 17 cases per group, totaling a sample size of 51 cases [19, 20, 21]. To control for potential biases arising from sample dropouts, an additional 20% increase in sample size was added to the estimate, resulting in a final target of 63 cases, with 21 cases allocated to each group.

### 2.3 Inclusion Criteria

(1) Unilateral stroke confirmed by computed tomography or magnetic resonance imaging [22]; (2) First-ever stroke occurring within 2 weeks to 6 months after onset; (3) Age ≥18 years; (4) Presence of penetration and/or aspiration confirmed by FEES [4]; (5) Water swallowing test grade ≥3; (6) Signed informed consent obtained from the patient or their family members.

### 2.4 Exclusion Criteria

(1) Dysphagia attributable to neurological conditions other than stroke, such as Parkinson’s disease, Alzheimer’s disease, traumatic brain injury, cerebellar or brainstem strokes, and head/neck tumors; (2) Pregnancy or lactation; (3) Acutely ill or medically unstable patients (e.g., unstable hemodynamics, active progressive illness); (4) Inability to comply with FEES, MEP, or swallowing function assessments due to cognitive, behavioral, or communication impairments; (5) Contraindications to transcranial magnetic stimulation (e.g., intracranial metal implants, epilepsy) or severe adverse reactions during previous rTMS sessions; (6) Disease progression or recurrent stroke during the trial; (7) Withdrawal of informed consent or unwillingness to continue participation.

### 2.5 Intervention Methods

During the treatment phase, to simulate the real-world scenario of multimodal integrated rehabilitation, all patients received standardized conventional swallowing rehabilitation therapy alongside their group-specific interventions. This conventional therapy consisted of a 2-week regimen administered once daily. The conventional rehabilitation included the following components: swallowing function training, oral sensory stimulation, acupuncture therapy, and postural compensatory training. Swallowing function training, oral sensory stimulation, and postural compensatory training were conducted by qualified rehabilitation therapists [3, 4, 5, 23]. Acupuncture treatment was performed by a certified acupuncturist. The acupoint selection protocol followed the principles of “local point selection, stage-specific treatment, and integration of pattern differentiation with disease diagnosis”. Based on these principles, differentiated acupoint prescriptions were designed to address the distinct stages of PSD (oral phase, pharyngeal phase, and oral-pharyngeal mixed phase) [23, 24]. Additionally, stimulation was delivered using a figure-of-8 coil connected to a MagNeuro 60 transcranial magnetic stimulator (Nanjing Vishee Medical Technology Co., Ltd., Nanjing, China). The sham rTMS group received bilateral sham stimulation over the swallowing cortex; affected rTMS group received real stimulation on the affected hemisphere combined with sham stimulation on the contralesional side; and bilateral rTMS group received real stimulation to both hemispheres. The rTMS parameters were set as follows: frequency of 10 Hz, intensity at 100% of the resting motor threshold (RMT), train duration of 2 s, inter-train interval of 10 s, and a total of 1200 pulses per session. Treatment was administered once daily, 7 days a week, for 2 weeks. Sham stimulation was performed using the flipped-coil method, where the figure-of-8 coil was rotated 180°, aligning the induced magnetic field tangentially to the scalp. This approach produced acoustic artifacts similar to real stimulation without delivering an effective magnetic field intracranially. Prior validation tests confirmed that participants could not reliably distinguish between real and sham stimulation. Immediately after the first treatment session, a blinding assessment was conducted by asking patients, “Do you believe you received real or sham stimulation in this session?” to evaluate their awareness of group assignment. Based on previous research indicating that the excitatory effects of rTMS can last approximately 30 minutes and remain effective over intermittent periods of 20–30 minutes, the bilateral rTMS group received stimulation to the unaffected hemisphere first, followed by the affected hemisphere, with the aim of achieving an additive effect. During treatment, patients were positioned comfortably in a supine or seated posture. A figure-of-eight coil was placed over the primary motor cortex representation area (swallowing cortical hotspot) of the suprahyoid muscles, as determined by MEP mapping. Therapists administered the corresponding stimulation according to group assignment. Treatment was immediately discontinued if patients experienced intolerable discomfort.

### 2.6 Evaluation Methods

This study systematically collected baseline demographic and clinical data from all enrolled patients prior to the trial commencement. Information included age, gender, stroke location, and stroke type, along with relevant outcome measures, to evaluate the comparability of baseline characteristics across groups. The primary and secondary outcome measures comprised the SSA, yale pharyngeal residue severity rating scale (YPR-SRS), and MEP. All assessments were conducted by a rehabilitation therapist blinded to the intervention at two time points: baseline (T0) and after two weeks of intervention (T1). Throughout the trial period, any adverse events (including seizures, headache, dizziness, syncope, dyspnea, local scalp/neck redness, and tinnitus-as well as participant dropouts) were meticulously documented to comprehensively evaluate treatment safety and tolerability. Prior to the trial, all assessors received standardized training on the relevant outcome measures to ensure consistency and accuracy in data collection.

#### 2.6.1 Primary Outcome Indicator

In this study, SSA was used as the primary outcome indicator. SSA is a simple, safe, and easily promotable tool for assessing swallowing function, demonstrating good sensitivity, specificity, reliability, and validity in the evaluation of swallowing disorders related to stroke and other conditions. The scale consists of three parts: clinical examination, a 5 mL water swallow test, and a 60 mL water swallow test, with a total score ranging from 18 to 46 points. A higher score indicates a more severe swallowing dysfunction. Multiple studies have shown that SSA scores have high consistency with gold standard results such as swallow imaging, effectively identifying the risk of swallowing disorders and associated complications, and possessing good clinical predictive value [25, 26].

#### 2.6.2 Secondary Outcome Indicators

The other measures, including the Penetration-Aspiration Scale (PAS) score and Yale Pharyngeal Residue Severity Rating Scale (YPR-SRS) score derived from FEES, as well as MEP parameters, were pre-defined as exploratory secondary outcome measures. The analysis of these measures was intended to generate preliminary evidence and hypotheses for future research.

2.6.2.1 PAS and YPR-SRS Based on FEES

FEES is one of the “gold standards” for swallowing function assessment. It allows for direct observation of the pharyngeal structures, food residue, and dynamic changes in swallowing by having patients swallow boluses of varying viscosities. This method has high sensitivity for detecting pharyngeal residue and can effectively assess the speed of swallowing initiation, pharyngeal clearance efficiency, and the degree of aspiration [9, 25, 27]. (1) The PAS scale is used to quantify the severity of airway penetration and aspiration, with a scoring range of 1 to 8, where a score of 8 represents silent aspiration. This scale has good sensitivity, specificity, and reliability and has been widely used in swallowing disorder research [26]. (2) The YPR-SRS scale is a five-point visual assessment tool focused on evaluating the location and amount of residue in the valleculae and piriform sinuses. A higher score indicates a greater amount of residue and lower pharyngeal clearance efficiency [27].

2.6.2.2 Localization of Motor Cortex Hotspots and Measurement of MEP for the Suprahyoid Muscles

MEP is a non-invasive neurophysiological testing technique mainly used to assess the function of the neural conduction pathways from the motor cortex to the muscles, including their overall synchrony and structural integrity [9, 26]. The analysis of MEP primarily relies on two indicators: amplitude and latency. The amplitude reflects the number and synchrony of the activated motor neurons, while the latency indicates the conduction velocity of the neural impulses. These indicators provide critical neurophysiological evidence for assessing the excitability and integrity of the corticospinal tract [14].

MEPs were measured in all patients before repetitive rTMS intervention to individualize the stimulation site and intensity. The specific procedure was as follows: after the patient assumed a comfortable position and relaxed fully, the operator placed the rTMS positioning cap and recording electrodes according to the International 10–20 electroencephalogram electrode placement system. The examiner then delivered a single-pulse stimulus to the primary motor cortex at 30% of the maximum output intensity. The stimulus intensity was gradually increased and the coil position was finely adjusted until the surface electrodes over the abductor pollicis brevis (APB) muscle recorded MEPs with the largest amplitude and good reproducibility; this location was identified as the cortical “hot spot” for the APB. Subsequently, at this “hot spot”, the stimulus intensity was progressively decreased [28]. The minimum intensity required to elicit MEPs with an amplitude exceeding 50 µV in at least 5 out of 10 consecutive stimuli was defined as the RMT [29].

After completing the above measurements, the recording electrode was placed on the suprahyoid muscle (Location: 2 cm lateral to the midpoint of the line connecting the middle of the hyoid bone and the midpoint of the mandible), the reference electrode was placed 2 cm lateral to the recording electrode, and the ground electrode was placed on the proximal forearm. The coil was positioned approximately 3 cm anterior to the vertex (Cz point) and about 7.5 cm laterally [30]. Single-pulse stimuli were delivered at 100% RMT intensity, and the coil was finely adjusted to locate the cortical hot spot for the suprahyoid muscle that could evoke consistent and stable MEP waveforms. Once the hot spot was identified, 10 consecutive stimuli were delivered. The five MEP waveforms with the best reproducibility were selected to calculate and record the RMT, latency, and amplitude for the suprahyoid muscle group. If no MEP could be elicited from the affected side, the corresponding data from the mirror region of the hot spot on the healthy side were used as a substitute [28, 30]. All operations were performed by the same professional neurodiagnostic physician, strictly adhering to MEP detection standards to ensure accurate localization of the swallowing-related cortex and the effectiveness of the therapeutic stimulation intensity (100% RMT).

### 2.7 Statistical Analysis

Statistical analyses were performed using SPSS 27.0 (IBM Corp., Armonk, NY, USA). Normality of continuous variables was assessed using the Shapiro-Wilk test and histograms. Normally distributed data are presented as mean ± standard deviation ($\bar{x}$ ± s), while non-normally distributed data are expressed as median (interquartile range) [M (P25, P75)]. Categorical data are reported as number (percentage). The primary outcome measure, SSA score, and continuous exploratory secondary outcomes (e.g., MEP latency and amplitude) were compared between groups using the Kruskal-Wallis test. Within-group comparisons were conducted using the Wilcoxon signed-rank test. For ordinal categorical variables, including PAS and YPR-SRS scores, between-group comparisons were also performed with the Kruskal-Wallis test, and within-group changes were analyzed with the Wilcoxon signed-rank test. If the overall between-group test was significant, post hoc pairwise comparisons were performed using Dunn’s test with Bonferroni correction. A linear mixed-effects model was fitted for SSA scores at T0 and T1, with group and time as fixed effects and subject as a random effect, and Bonferroni correction was applied. Furthermore, the change in SSA score from baseline to the end of treatment (ΔSSA = SSA_T₀_ – SSA_T₁_) was calculated to assess the degree of swallowing function improvement. Based on previous literature, a ΔSSA ≥4 points was set as an exploratory threshold to define treatment response [30, 31]. The response rate was determined accordingly, and a chi-square test was used to compare response rates between groups. Effect sizes are expressed as Cohen’s d (0.20, 0.50, and 0.80 corresponding to small, medium, and large effects, respectively) [32, 33]. All statistical tests were two-tailed, and *p* < 0.05 was considered statistically significant.

## 3. Results

### 3.1 Analysis of General Information

**Fig. 1.**
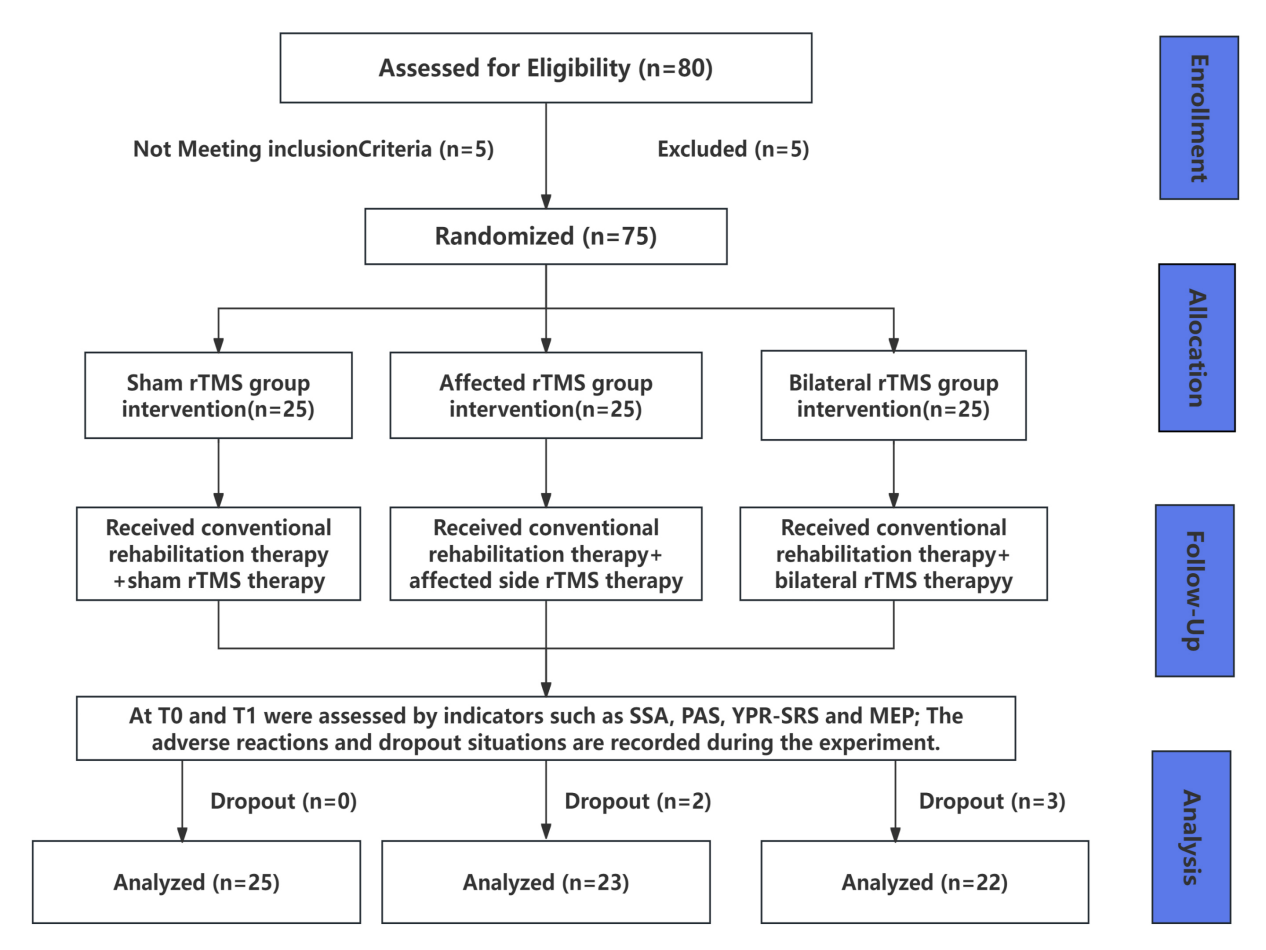
**Flowchart of the experiment**. rTMS, Repetitive transcranial magnetic stimulation; SSA, Standardized swallowing assessment; PAS, Penetration aspiration scale; YPR-SRS, yale pharyngeal residue severity rating scale; MEP, Motor evoked potential. T0, Baseline before the intervention; T1, 2 weeks after rTMS intervention.

Between March 2023 and October 2025, a total of 75 patients with PSD were enrolled in this study. During the trial, five patients were lost to follow-up: two in the affected rTMS group withdrew one week after intervention due to conflicts with other treatment schedules, and three in the bilateral rTMS group dropped out before completing one week of intervention due to hospital discharge or personal reasons. Ultimately, 70 patients completed all scale assessments, FEES, and MEP data collection, and were included in the statistical analysis. The detailed trial flow is illustrated in Fig. 1. Analysis of baseline characteristics showed no statistically significant differences among the three groups in terms of age, sex, disease duration, stroke location, or stroke type (*p* > 0.05). Furthermore, no significant intergroup differences were observed at baseline in SSA scores, PAS scores, pharyngeal residue scores, or MEP parameters (*p* > 0.05), indicating that the baseline characteristics were well balanced across groups (Table 1).

**Table 1.** **Comparison of patient data at T0 among the 3 groups (n = 70)**.

Variables			Sham rTMS	Affected rTMS	Bilateral rTMS	Statistics	*p*-value
			(n = 25)	(n = 23)	(n = 22)		
Age (years, $\bar{x}$ ± s)			61.800 ± 12.196	65.570 ± 13.777	57.590 ± 13.504	2.070	0.134
Gender							
	Male		16	15	15	0.095	0.954
	Female		9	8	7		
Time since stroke onset (days, mean $\bar{x}$ ± s)			83.84 ± 44.10	82.70 ± 53.95	86.64 ± 50.21	0.166	0.920
Stroke types							
	Ischemic		14	17	17	2.911	0.233
	Hemorrhagic		11	6	5		
Stroke sites							
	Left		10	13	13	2.063	0.356
	Right		15	10	9		
T0							
SSA ($\bar{x}$ ± s)			32.960 ± 4.937	33.570 ± 4.804	32.000 ± 5.372	0.551	0.579
PAS			4 (3, 5)	4 (3, 6)	4 (3, 5)	0.437	0.804
Md (P25, P75)							
Oral residual scores							
Vallecular residue			4 (4, 4)	4 (4, 4)	4 (3, 4)	1.050	0.592
Md (P25, P75)							
Pyriform sinus residue			4 (3, 4)	4 (3, 4)	4 (4, 4)	1.555	0.457
Md (P25, P75)							
MEP							
	Amplitude						
		Ipsilesional ($\bar{x}$ ± s)	160.2 ± 44.76	173.5 ± 38.24	157.5 ± 43.89	2.308	0.315
		Contralesional ($\bar{x}$ ± s)	131.9 ± 37.83	140.3 ± 34.26	127.7 ± 38.00	1.594	0.451
	Latencyperiod						
		Ipsilesional ($\bar{x}$ ± s)	4.941 ± 1.037	4.813 ± 0.196	4.943 ± 0.492	0.474	0.789
		Contralesional ($\bar{x}$ ± s)	5.000 ± 0.324	4.948 ± 0.458	5.409± 0.757	5.258	0.072

### 3.2 Comparison of SSA Scores Among the Three Groups

**Fig. 2.**
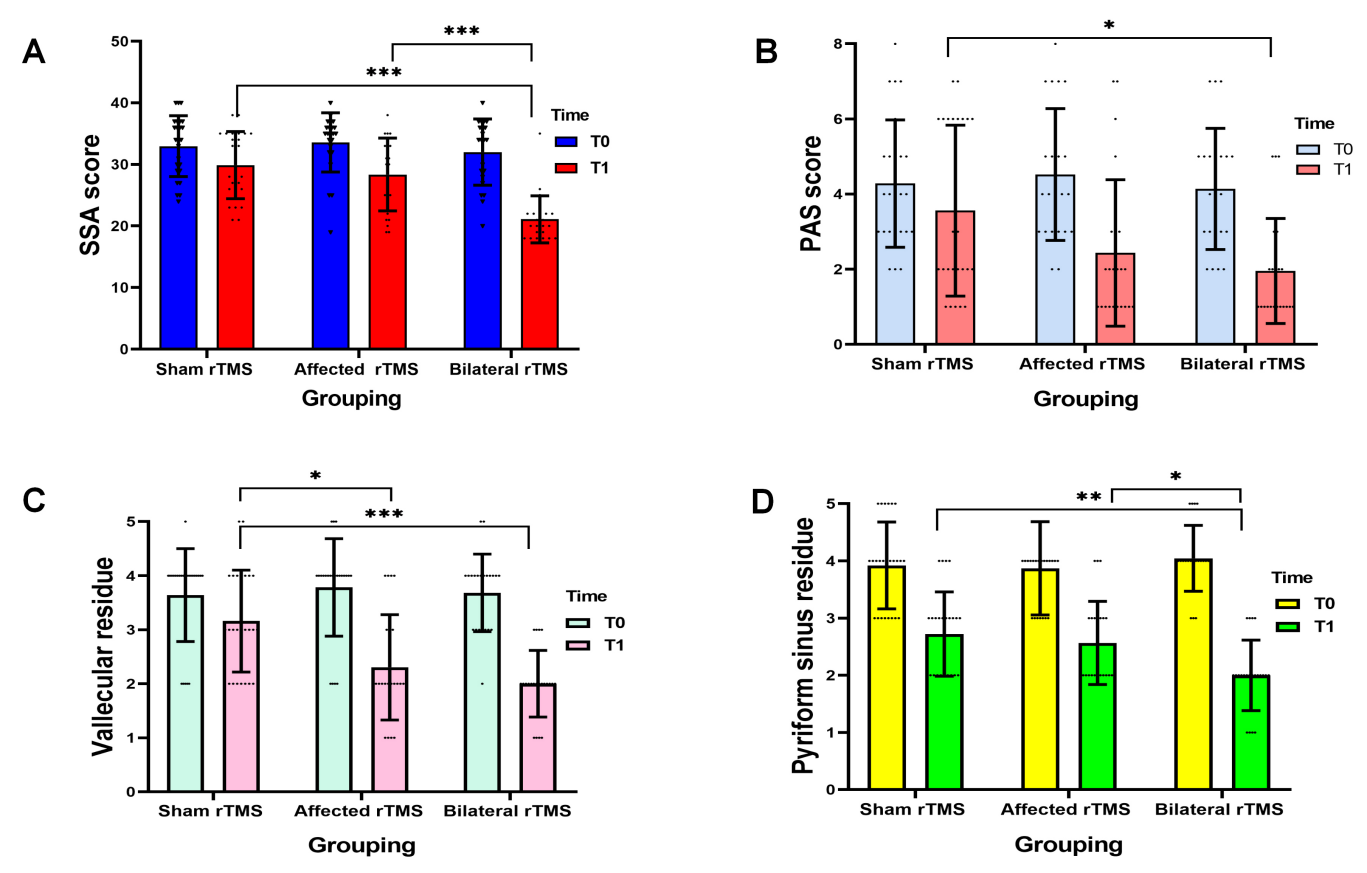
**Comparison of SSA scores (A), PAS scores (B), vallecular residue scores (C), and pyriform sinus residue scores (D) before and after treatment in the three groups**. ^*^p < 0.05; ^**^p < 0.01; ^*⁣**^p < 0.001.

The SSA scores of the three groups at T0 and T1 are presented in Table 2 and Fig. 2A. Overall, the SSA scores improved significantly in all groups after treatment, with T1 scores being significantly lower than those at T0 (*p* < 0.001). Further between-group comparisons revealed that at T1, the SSA score in the bilateral rTMS group was significantly lower than that in both the sham stimulation group and the affected rTMS group (*p* < 0.001).

**Table 2.** **Comparison of the SSA, PAS, YPR-SRS, and MEP scores before and after treatment in the 3 groups**.

Variables				Sham rTMS	Affected rTMS	Bilateral rTMS	*p*-value
				(n = 25)	(n = 23)	(n = 22)	
SSA							
	T0 ($\bar{x}$ ± s)			32.960 ± 4.937	33.570 ± 4.804	32.000 ± 5.372	0.579
		95% CI		(30.92, 35.00)	(31.49, 35.64)	(29.62, 34.38)	
	T1 ($\bar{x}$ ± s)			29.880 ± 5.457	28.350 ± 5.913	21.090 ± 3.829	<0.001
		95% CI		(27.63, 32.13)	(25.79, 30.90)	(19.39, 22.79)	
	*p*-value			<0.001	<0.001	<0.001	
	Change value (ΔSSA)			3.080 ± 2.768	5.217 ± 5.143	10.910 ± 6.346	<0.001
	Effective rate (ΔSSA ≥4)			32.00%	47.83%	77.27%	0.008
PAS							
	T0 Md (P25, P75)			4 (3, 5)	4 (3, 6)	4 (3, 5)	0.804
		95% CI		(3, 5)	(3, 5)	(3, 5)	
	T1 Md (P25, P75)			2 (2, 6)	2 (1, 3)	1 (1, 2.25)	0.017
		95% CI		(2, 6)	(1, 3)	(1, 2)	
	*p*-value			0.126	<0.001	<0.001	
	PAS ≤2, n (%)						
		T0		3 (12.0%)	3 (13.0%)	4 (18.2%)	0.815
		T1		13 (52.0%)	16 (69.6%)	17 (77.3%)	0.170
	PAS ≤5, n (%)						
		T0		20 (80.0%)	17 (73.9%)	19 (86.4%)	0.580
		T1		21 (84.0%)	20 (87.0%)	21 (95.5%)	0.448
Vallecular residue							
	T0 Md (P25, P75)			4 (4, 4)	4 (4, 4)	4 (3, 4)	0.592
		95% CI		(4, 4)	(4, 4)	(3, 4)	
	T1 Md (P25, P75)			3 (2, 4)	2 (2, 3)	2 (2, 2)	<0.001
		95% CI		(3, 4)	(2, 3)	(2, 2)	
	*p*-value			0.037	<0.001	<0.001	
Pyriform sinus residue							
	T0 Md (P25, P75)			4 (3, 4)	4 (3, 4)	4 (4, 4)	0.457
		95% CI		(3, 4)	(3, 4)	(4, 4)	
	T1 Md (P25, P75)			2 (2, 3)	2 (2, 3)	2 (2, 2)	0.004
		95% CI		(2, 3)	(2, 3)	(2, 2)	
	*p*-value			<0.001	<0.001	<0.001	
MEP Amplitude							
	Ipsilesional						
		T0 ($\bar{x}$ ± s)		160.20 ± 44.76	173.50 ± 38.24	157.50 ± 43.89	0.315
			95% CI	(141.7, 178.7)	(156.9, 190.0)	(138.0, 176.9)	
		T1 ($\bar{x}$ ± s)		269.20 ± 88.55	275.50 ± 49.89	314.40 ± 61.26	0.023
			95% CI	(232.7, 305.8)	(253.9, 297.1)	(287.2, 341.6)	
		*p*-value		<0.001	<0.001	<0.001	
		Effect size (D)		2.435	2.295	2.944	
	Contralesional						
		T0 ($\bar{x}$ ± s)		131.90 ± 37.83	140.30 ± 34.26	127.70 ± 38.00	0.451
			95% CI	(116.3, 147.6)	(125.4, 155.1)	(110.8, 144.5)	
		T1 ($\bar{x}$ ± s)		217.30 ± 50.73	208.50 ± 59.70	267.30 ± 65.01	0.007
			95% CI	(196.4, 238.3)	(182.4, 234.5)	(238.5, 296.1)	
		*p*-value		<0.001	<0.001	<0.001	
		Effect size (D)		1.908	1.391	2.622	
Latency period							
	Ipsilesional						
		T0 ($\bar{x}$ ± s)		4.941 ± 1.037	4.813 ± 0.196	4.943 ± 0.492	0.789
			95% CI	(4.513, 5.369)	(4.728, 4.898)	(4.725, 5.161)	
		T1 ($\bar{x}$ ± s)		4.776 ± 0.249	4.713 ± 0.212	4.845 ± 0.250	0.196
			95% CI	(4.673, 4.879)	(4.621, 4.805)	(4.735, 4.956)	
		*p*-value		0.593	0.079	0.873	
		Effect size (D)		0.219	0.490	0.251	
	Contralesional						
		T0 ($\bar{x}$ ± s)		5.000 ± 0.324	4.948 ± 0.458	5.409 ± 0.757	0.789
			95% CI	(4.866, 5.134)	(4.750, 5.146)	(5.073, 5.745)	
		T1 ($\bar{x}$ ± s)		4.912 ± 0.222	4.835 ± 0.206	4.957 ± 0.224	0.061
			95% CI	(4.820, 5.004)	(4.746, 4.924)	(4.858, 5.056)	
		*p*-value		0.270	0.486	0.046	
		Effect size (D)		0.317	0.318	0.810	

Effect sizes of 0.2, 0.5, and 0.8 correspond to small, medium, and large, respectively.

To systematically quantify the independent and interactive effects of group and time on SSA scores, a linear mixed-effects model was constructed based on data from patients who completed the full intervention. The model results were as follows: a significant main effect of group (F = 54.920, *p* < 0.001), indicating that the overall SSA scores differed significantly among the three groups from baseline to follow-up; a significant main effect of time (F = 12.640, *p* < 0.001) suggesting a substantial overall change in SSA scores over time after intervention; and a significant group × time interaction (F = 7.230, *p* = 0.001), demonstrating statistically different trends in SSA score changes before and after treatment among the groups (Table 3).

**Table 3.** **Results of linear mixed effect model analysis of SSA**.

Model effect	F (DFn, DFd)	*p*-value
Between-group main effect	54.920 (2, 134)	<0.001
Time main effect	12.640 (1, 134)	<0.001
Group × Time interaction	7.230 (2, 134)	0.001

**Fig. 3.**
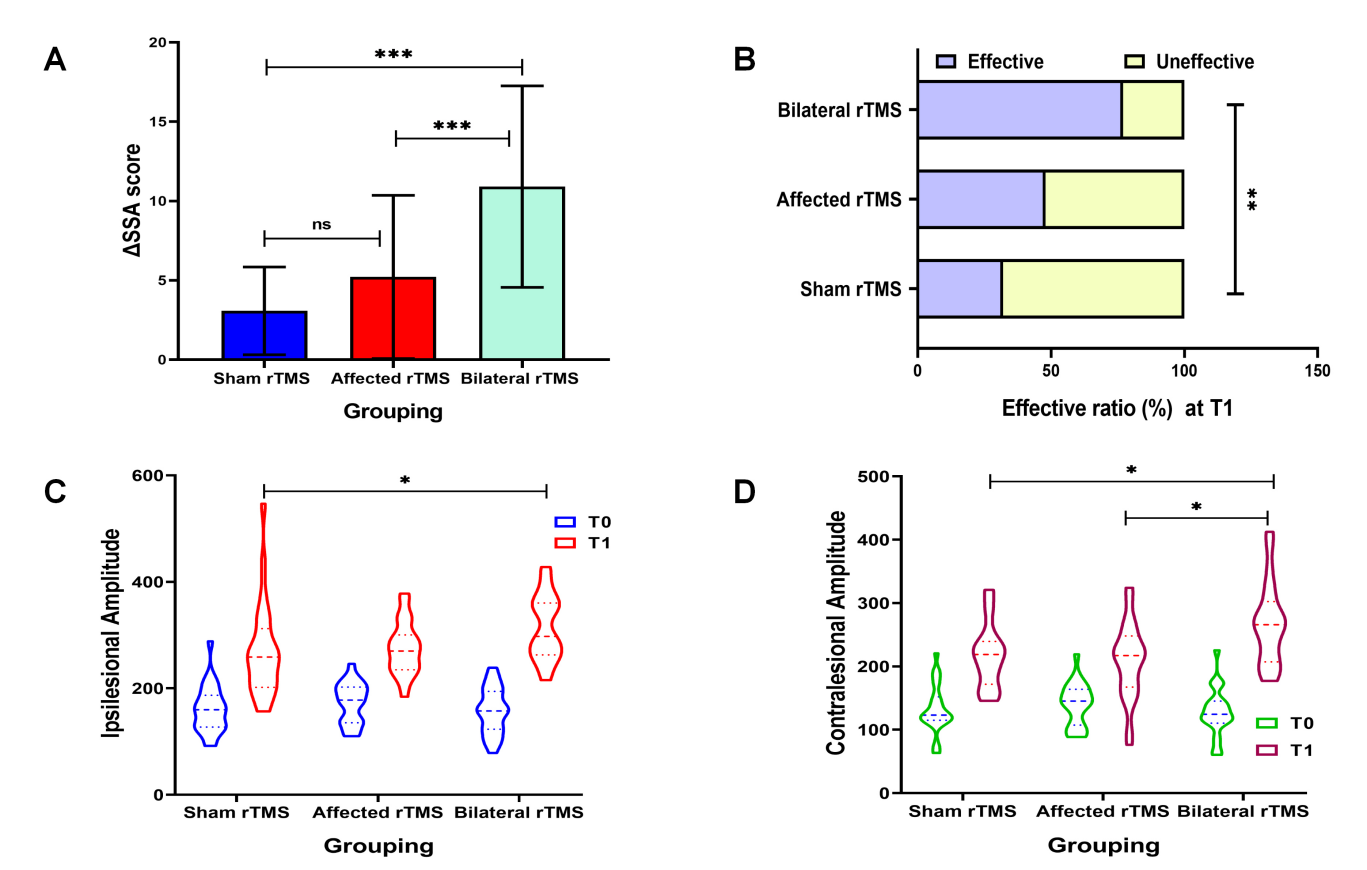
**Comparison of the ΔSSA (A), SSA effective ratio (B), MEP amplitudes in the ipsilesional (C) and contralesional (D) regions among the three groups**. ns, not significant; ^*^p < 0.05; ^**^p < 0.01; ^*⁣**^p < 0.001.

To further evaluate the magnitude of improvement in swallowing function, the change in SSA score (ΔSSA) was calculated. The results showed that the ΔSSA in the bilateral rTMS group was significantly greater than that in both the sham group and affected rTMS group (*p* < 0.001), indicating a more pronounced improvement in swallowing function in the bilateral rTMS group (Fig. 3A). The response rate, defined as the proportion of patients achieving a ΔSSA of at least 4 points, was 77.27% in the bilateral rTMS group, which was significantly higher than the rates in the sham group (32.00%) and the affected rTMS group (47.83%), with a statistically significant between-group difference (χ^2^ = 9.780, *p* = 0.008) (Table 2, Fig. 3B).

Additionally, an intention-to-treat analysis was conducted, applying the baseline observation carried forward (BOCF) method to the five patients who dropped out. The results showed that SSA scores still differed significantly among the three groups after treatment (*p* < 0.001). Further pairwise comparisons confirmed that the SSA score in the bilateral rTMS group remained significantly lower than that in both the sham stimulation group and the affected-side rTMS group (*p* < 0.01). A mixed-effects model constructed on this basis continued to support the above conclusions, yielding the following results: a significant main effect of group (F = 46.67, *p* < 0.001); a significant main effect of time (F = 9.406, *p* = 0.0001); and a significant group × time interaction (F = 5.232, *p* = 0.006). Detailed data are provided in **Supplemental Material**.

### 3.3 Comparison of PAS Scores Among the Three Groups

The PAS scores of the three groups of patients at T0 and T1 are shown in Table 2 and Fig. 2B. Statistical analysis revealed that the PAS scores in the affected rTMS group and the bilateral rTMS group at T1 were significantly lower than those at T0 (*p* < 0.001). However, the difference in scores for the sham rTMS group before and after treatment was not statistically significant (*p* = 0.126). Inter-group comparisons showed a significant overall difference in PAS scores among the three groups at T1 (*p* = 0.017). Further pairwise comparisons indicated that the PAS score of the bilateral rTMS group was significantly lower than that of the sham rTMS group (*p* = 0.017).

Furthermore, the proportion of patients achieving safe swallowing (PAS ≤2, indicating penetration without aspiration) increased significantly: from 12% to 52% in the sham group, from 13% to 69.6% in the affected rTMS group, and from 18.2% to 77.3% in the bilateral rTMS group. The proportion achieving basic safe swallowing (PAS ≤5, indicating that aspirated material can be cleared) also improved: from 80% to 84% in the sham group, from 73.9% to 87.0% in the affected rTMS group, and from 86.4% to 95.5% in the bilateral rTMS group.

### 3.4 Comparison of YPR-SRS Scores Among the Three Groups

Table 2 and Fig. 2C,D summarize the results of the vallecular residue and pyriform sinus residue scoring for the three groups of patients at T0 and T1. Overall, the residue scores for both the vallecula and the pyriform sinus showed significant improvement over time, with scores at T1 being significantly lower than those at T0 (*p* < 0.05). Inter-group comparisons indicated statistically significant overall differences in vallecular residue scores (*p* < 0.001) and pyriform sinus residue scores (*p* = 0.004) among the three groups at T1. Further pairwise comparisons revealed that for vallecular residue, the scores for the affected rTMS group and the bilateral rTMS group were both significantly lower than that of the sham rTMS group (*p* < 0.05). Regarding pyriform sinus residue, the score for the bilateral rTMS group was significantly lower than those for the affected rTMS group and the sham rTMS group (*p* < 0.05).

### 3.5 Comparison of MEP Parameters Among the Three Groups

The MEP parameters for the three groups at T0 and T1 are summarized in Table 2 and Fig. 3C,D. Overall, the MEP amplitude significantly increased from T0 to T1 in all groups (*p* < 0.001). Regarding MEP latency, a statistically significant difference between pre-and post-treatment values was observed only on the unaffected side in the bilateral rTMS group (*p* = 0.046). Inter-group comparisons at T1 revealed significant differences in MEP amplitude among the groups, both on the ipsilesional side (*p* = 0.023) and the contralesional side (*p* = 0.007), whereas no significant inter-group differences were found in latency (*p* > 0.05). Further pairwise comparisons showed that the MEP amplitude on the unaffected side in the bilateral rTMS group was significantly higher than that in the sham group (*p* = 0.020). Moreover, the MEP amplitude on the affected side in the bilateral rTMS group was not only significantly higher than that in the sham stimulation group (*p* = 0.026) but also significantly higher than that in the affected-side rTMS group (*p* = 0.013).

### 3.6 Effect Size

At T1, this study calculated the Cohen’s D values for the changes in functional outcomes to assess the effect sizes of different indicators, with the effect sizes for each group summarized as follows (Table 2):

Sham rTMS Group: SSA score improvement demonstrated a medium effect size (D = 0.592); MEP amplitude showed large effects on both the unaffected side and affected side (D = 2.435 and 1.908, respectively); Changes in MEP latency were classified as a small effect (unaffected side D = 0.219; affected side D = 0.317).

Affected rTMS Group: SSA score improvement showed a large effect size (D = 0.969); MEP amplitude on both the unaffected side and affected side also exhibited large effects (D = 2.295 and 1.391, respectively); Changes in MEP latency remained a small effect (unaffected side D = 0.490; affected side D = 0.318).

Bilateral rTMS Group: SSA score improvement resulted in a large effect size (D = 2.339); MEP amplitude on both the unaffected side and affected side demonstrated large effects (D = 2.944 and 2.622, respectively); Notably, the MEP latency on the affected side achieved a large effect size (D = 0.810), while the change in latency on the unaffected side remained a small effect (D = 0.251).

### 3.7 Blinding Assessment

The blinding assessment conducted after the first treatment session revealed that the proportion of patients who correctly identified their actual group assignment was 68% in the sham group, 60% in the affected rTMS group, and 76% in the bilateral rTMS group. A chi-square test showed no statistically significant difference in the accuracy rates among the three groups (χ^2^ = 1.471, *p* = 0.479). These results indicate that the blinding method was effective, and patients could not reliably distinguish between real and sham stimulation, confirming the successful implementation of blinding in this study.

### 3.8 Complications and Adverse Reactions

All patients successfully completed the treatment, with no cases of dropout due to intolerance, indicating overall good tolerability. During the study, a total of 5 cases of transient scalp discomfort were reported (1 case in the sham rTMS group, 2 cases in the affected rTMS group, and 2 cases in the bilateral rTMS group). Symptoms were alleviated after pausing stimulation and taking a short break. No other serious adverse events, such as headache, epilepsy, tinnitus, or psychological discomfort, were observed.

## 4. Discussion

This randomized controlled trial investigated the efficacy, safety, and underlying neural mechanisms of 10 Hz rTMS applied to the affected-side or bilateral swallowing cortical regions, combined with conventional rehabilitation, for functional recovery in patients with PSD. Utilizing a multidimensional assessment framework encompassing behavioral and neurophysiological outcomes, the study results indicate that: (1) while rTMS combined with conventional rehabilitation (regardless of stimulation target) effectively improved swallowing function in PSD patients, the bilateral rTMS group demonstrated superior comprehensive efficacy, showing significantly greater improvements in overall swallowing efficiency, reduction of aspiration risk, and decrease in pharyngeal residue compared to both the sham and affected rTMS groups; (2) neurophysiological data revealed that all interventions enhanced cortical excitability in swallowing-related areas, and the bilateral rTMS paradigm not only augmented excitability but also potentially optimized the conduction efficiency of neural pathways; (3) effect size analysis further supported these findings, with bilateral rTMS showing a “large effect” for improvement in the primary functional outcome; (4) regarding safety, no serious adverse events occurred with any intervention protocol, and all were well tolerated, confirming the safety of 10 Hz rTMS in this clinical application.

### 4.1 Mechanisms of PSD

Swallowing function involves multi-stage coordinated activities from the oral cavity to the esophagus, relying on a complex neural network regulated by bilateral cerebral cortices, subcortical structures, the brainstem, and the cerebellum [31, 33]. Within this network, the bilateral cerebral hemispheres jointly control swallowing via corticofugal projections, typically with one hemisphere serving as the dominant side and the other playing a synergistic and compensatory role [11, 34]. When stroke injures key nodes of this network-such as the primary motor cortex, anterior insula, anterior cingulate cortex, or frontal operculum-or disrupts the white matter pathways connecting them, the integrity and coordination of the network are compromised. The core pathological mechanisms involve desynchronization of cortico-subcortical neural circuits and impaired neuromuscular control of muscles such as the suprahyoid group, ultimately leading to clinical symptoms including pharyngeal residue, aspiration, and delayed swallowing initiation [3, 35, 36]. This understanding of network dysfunction provides a theoretical basis for the use of neuromodulation techniques, such as rTMS, to target and repair neural circuits.

### 4.2 Theoretical Models and Target Selection for rTMS Intervention

The bilateral rTMS intervention strategy adopted in this study was primarily based on the theoretical frameworks of the interhemispheric competition model and the dual-mode balance recovery model [9, 11, 17, 18]. Following stroke, the excitability of the affected hemisphere decreases, while the unaffected hemisphere may become hyperactive due to loss of interhemispheric inhibition, thereby impeding the recovery of swallowing function [12]. In contrast to conventional unilateral intervention approaches, bilateral high-frequency rTMS (e.g., 10 Hz) simultaneously modulates excitability in both hemispheres: it promotes functional reorganization in the affected hemisphere while suppressing excessive activity in the unaffected hemisphere, ultimately restoring the overall function of the swallowing network. As swallowing is a physiologically bilateral cortical process, its neural network relies on compensatory mechanisms from the unaffected hemisphere after unilateral damage [37, 38, 39]. Thus, bilateral synchronous stimulation may activate potential compensatory pathways, yielding synergistic effects. Although there is no unified standard for optimal rTMS parameters in PSD treatment, high-frequency stimulation (≥5 Hz) applied to bilateral swallowing cortices (e.g., the suprahyoid motor cortex) at an intensity of 80%–120% RMT represents a common strategy [15, 19, 21, 40]. This study employed a 10 Hz bilateral protocol to enhance cortical excitability and long-term plasticity (LTP) plasticity, thereby optimizing neuroplasticity and providing a theoretical basis for multi-target neuromodulation strategies.

### 4.3 Clinical Efficacy Advantages of Bilateral rTMS

The findings of this study clearly demonstrate that bilateral high-frequency rTMS offers significant advantages in improving swallowing function in patients with PSD. Specifically: Comprehensive improvement in swallowing function: The bilateral rTMS group showed significantly greater improvements in the SSA score (reflecting overall swallowing ability), PAS score (reflecting swallowing safety), and pharyngeal residue score (reflecting swallowing efficiency) compared to both the sham stimulation group and the unilateral rTMS group. High clinical response rate: Using a ΔSSA ≥4 points was set as an exploratory threshold to define treatment response, the response rate in the bilateral rTMS group reached 77.27%, significantly higher than the other two groups, with an effect size indicating a “large effect”, underscoring the clinical value of this intervention. Thus, these behavioral results align with previous studies, confirming that the bilateral rTMS strategy holds superior advantages in enhancing swallowing function, improving safety, and clearing efficiency, thereby offering a comprehensive approach to addressing the multifaceted challenges of PSD and providing strong evidence for clinical application [41, 42, 43, 44].

### 4.4 Neuroelectrophysiological Mechanisms of rTMS in Promoting Swallowing Function Recovery

Changes in MEPs provide an objective neurophysiological metric for elucidating the mechanisms of rTMS. The results of this study showed that the MEP amplitude significantly increased from baseline in all intervention groups after treatment, suggesting that conventional rehabilitation combined with rTMS may effectively enhance cortical excitability in swallowing-related areas. Notably, the bilateral rTMS group demonstrated a unique advantage: the improvement in MEP amplitude on the affected side was significantly greater than that in the affected-side rTMS group, indicating that bilateral stimulation may more effectively facilitate the recruitment and synchronized firing capacity of neural pathways in the affected hemisphere, potentially by modulating interhemispheric interactions [13, 14]. Particularly important is that only the bilateral rTMS group showed a significant shortening of MEP latency on the unaffected side. Given that latency reflects the neural conduction velocity from the cortex to the target muscles, improvement in this measure suggests that bilateral rTMS not only increased cortical excitability but may also have enhanced the conduction speed of swallowing-related neural pathways, possibly by optimizing myelination or synaptic transmission efficiency [45, 46, 47]. The underlying mechanisms may involve rTMS-induced modulation of neurotransmitter systems (e.g., GABA and glutamate) and upregulation of brain-derived neurotrophic factor expression, as observed in other studies [47, 48]. Therefore, bilateral rTMS may promote neural remodeling and functional recovery related to swallowing through a dual mechanism of “enhancing excitability” and “optimizing conductivity”.

### 4.5 Safety and Tolerability

All patients completed the treatment without any dropouts due to intolerance, indicating good overall tolerability of the intervention protocols across all groups. A total of five cases of transient scalp discomfort were reported among the three groups, with no significant difference in incidence rates between groups. Symptoms resolved after briefly pausing stimulation and allowing a short rest. No serious adverse events such as headache, seizures, tinnitus, or psychological discomfort were observed. These findings are consistent with the majority of reported studies, further supporting the favorable safety profile of rTMS in treating post-stroke dysphagia [49]. In this study, bilateral rTMS did not increase the risk of adverse reactions, demonstrating that both dual-target combined intervention and single intervention modalities possess reliable safety and clinical applicability [44].

## 5. Limitations and Future Directions

This study has several limitations. First, the single-center design and the lack of subgroup analyses based on stroke type, location, or severity (SSA or FEES) may limit the generalizability of the findings. Future research should employ multicenter, large-sample randomized controlled trials incorporating machine learning-based patient stratification to further validate the efficacy of bilateral rTMS in PSD. Second, the 2-week intervention period without a follow-up assessment precludes evaluation of long-term effects. Subsequent studies should consider extending the intervention duration and incorporating follow-ups at 4, 8, and 12 weeks post-intervention (e.g., via telephone-administered SSA combined with on-site FEES or MEP assessments) to determine the persistence of therapeutic effects and neuroplastic changes. Furthermore, while MEP was used to localize stimulation hotspots, the absence of MRI neuronavigation may have affected targeting precision. Although MEP can reflect cortical excitability, its utility in elucidating underlying mechanisms remains limited. Future investigations should integrate multimodal techniques such as fNIRS, fMRI, and biomarkers to provide deeper insights into the neural mechanisms of swallowing function recovery.

It should be noted that studies have shown that acupuncture exerts independent effects on the central nervous system. In the present study, acupuncture was included as a fixed component of conventional rehabilitation therapy to simulate real-world clinical practice [24]. Consequently, the current study design does not allow for a precise delineation of the individual contributions of acupuncture and rTMS to the observed therapeutic outcomes. To more purely evaluate the incremental benefit of rTMS on top of conventional rehabilitation, future studies could consider a more rigorous three-arm trial design.

## 6. Conclusions

This study demonstrates that conventional rehabilitation combined with 10 Hz rTMS targeting the swallowing cortex effectively improves PSD, with the bilateral rTMS strategy yielding superior therapeutic outcomes. Specifically, bilateral rTMS significantly enhanced swallowing efficiency, swallowing safety, and pharyngeal residue clearance, with a large effect size observed for the primary functional outcome. The underlying mechanism may involve simultaneous enhancement of cortical excitability and optimization of neural conduction velocity, which collectively promote functional improvement and neuroplastic remodeling of swallowing-related neural pathways. All treatment regimens were well tolerated, with no severe adverse events reported, confirming their safety profile. In summary, bilateral 10 Hz rTMS, as a multi-target neuromodulation strategy that synchronously modulates the swallowing cortices of both hemispheres, demonstrates clear clinical benefits and holds promise for broader application. Future research should extend the intervention period, conduct multicenter large-sample randomized controlled trials, and integrate multimodal neuroimaging techniques to further validate its long-term efficacy and elucidate its neural mechanisms in greater depth.

## Data Availability

The data that support the findings of this study are available on request from the corresponding author. The data are not publicly available due to privacy or ethical restrictions.

## Abbreviations

PSD, Post-stroke dysphagia; WST, Water swallowing test; FEES, Fiberoptic endoscopic evaluation of swallowing; rTMS, Repetitive transcranial magnetic stimulation; SSA, Standardized swallowing assessment; PAS, Penetration aspiration scale; MEP, Motor evoked potential.

## Supplementary Material

Supplementary material associated with this article can be found, in the online version, at https://doi.org/10.31083/RN49912.
