# Supplementary material for: Efficacy and Neurophysiological Mechanisms of 10 Hz Repetitive Transcranial Magnetic Stimulation for Post-Stroke Dysphagia: A Randomized Controlled Trial
Source: Rev Neurol. 2026 Apr 22;81(4):49912. doi: 10.31083/RN49912 (PMC13129558; doi:10.31083/RN49912)
Supplement: Supplementary file 1 [file 1576-6578-81-4-49912-s1.zip › Supplementary Material.docx]

| **Table1 . Results of intention-to-treat analysis and mixed-effects model for SSA scores among the three groups.** | | | | |
| --- | --- | --- | --- | --- |
| **Variables** | **Sham rTMS**  **(n=25)** | **Affected rTMS**  **(n=25)** | **Bilateral rTMS**  **(n=25)** | **p-value** |
| **SSA** |  |  |  |  |
| **T0**  ( ± s) | 32.960 ± 4.937 | 33.570 ± 4.804 | 32.000 ± 5.372 | 0.579 |
| 95% CI | (30.92, 35.00) | (31.49, 35.64) | (29.62, 34.38) |  |
| **T1**  ( ± s) | 29.88 ± 5.457 | 28.64 ± 5.758 | 22.52 ± 5.386 | ＜ 0.001 |
| 95% CI | (27.63, 32.13) | (26.26, 31.02) | (20.30, 24.74) |  |
| **p-value** | ＜ 0.001 | ＜ 0.001 | ＜ 0.001 |  |
| **Mixed-effects model** | | | | |
| **Model effect** | **F (DFn, DFd)** | | **p-value** | |
| **Between-group main effect** | 46.67 (1, 144) | | < 0.001 | |
| **Time main effect** | 9.406 (2, 144) | | 0.0001 | |
| **Group × Time interaction** | 5.232 (2, 144) | | 0.006 | |
| rTMS, Repetitive transcranial magnetic stimulation; SSA, Standardized swallowing assessment. | | | | |

**Supplemental Material**
